# From Signal Quality to Representation Stability in Textile Bioelectronic Interfaces

**DOI:** 10.34133/bmef.0325

**Published:** 2026-10-08

**Authors:** Li Guo

**Affiliations:** Swedish School of Textiles, University of Borås, Borås, Sweden.

## Abstract

Textile bioelectronic interfaces are emerging as wearable platforms for continuous physiological sensing, rehabilitation, and human–machine interaction. By integrating functional materials into textiles, electrodes and sensors can become part of the garment itself, forming a deformable interface with the body. Using surface electromyography (sEMG) acquired through textile-integrated electrodes as an illustrative case, this perspective argues that measured sEMG signals are shaped not only by muscle electrical activity but also by the deformable body–textile–electrode interface, whose state may change during use. Current evaluation of textile bioelectronic interfaces is often output centered, focusing on signal quality and downstream performance, which are necessary but insufficient for assessing whether measured signal patterns remain stable and reliable outside controlled conditions. This perspective proposes representation stability as a complementary evaluation criterion within an interface-centered framework. Representation stability asks whether measured signal patterns remain comparable as the physical sensing interface changes during real use. Within this framework, interface conditions become explicit test variables, with representation stability assessed using pre-specified criteria. Introducing representation stability extends evaluation from an output-centered toward an interface-centered paradigm. This could provide a more systematic basis for understanding, comparing, and ultimately designing textile bioelectronic interfaces that remain reliable under real-use variability.

## Background of Soft and Textile Bioelectronic Interfaces

Soft bioelectronics is moving toward mechanically compliant, skin-like, and wearable systems for continuous health monitoring, rehabilitation, and human–machine interaction [1,2]. Within this broader shift, textiles represent a distinct class, combining conformability and suitability for long-term wear with scalable integration of functional materials [3]. In this perspective, textile bioelectronic interfaces refer to textile-based or textile-integrated systems in which conductive or embedded functional elements form the body-contacting interface for physiological sensing, stimulation, or interaction. Textiles act as electrodes, sensors, interconnects, and distributed bioelectronic interfaces whose structure and mechanics shape electrical contact and signal acquisition [3,4].

In surface electromyography (sEMG), textile-integrated electrodes offer a promising route toward long-term sensing beyond controlled settings [4,5]. When electrodes are integrated into textiles, sensing occurs through a deformable body–textile–electrode interface. The measured sEMG signal is shaped by both muscle electrical activity and the state of this interface, including contact pressure, deformation, electrode displacement, and electrode–skin impedance, which may change during use. The deformable interface therefore becomes part of the measurement problem: changes in its state may alter the measured signal pattern even when the same motor task is performed in a comparable way.

## Progress and Limitations of Current Evaluation

Current evaluation of textile-integrated sEMG systems focuses on 2 approaches: electrode and signal quality assessment, using measures such as amplitude, signal-to-noise ratio, and comparison with Ag/AgCl electrodes [4,5], and, in control-oriented systems, downstream task-performance assessment such as classification or control accuracy [6]. In both approaches, variation in the interface is often treated as experimental noise to be minimized [4,7]. In real use, however, such variation naturally occurs as a deformable textile interface conforms to and moves with the body, making it a condition to be characterized rather than simply minimized.

Good signal quality or task performance in one interface condition does not necessarily mean that the measured signal pattern remains stable when the interface condition changes. Changes in fit or electrode displacement may alter the signal pattern without eliminating the signal itself. Similar effects have been reported in myoelectric control, where limb position and electrode shift alter extracted features and reduce classification performance [8]. In such cases, the problem is deeper than whether the signal remains strong, clean, or classifiable: conventional metrics may confirm signal quality or task performance without revealing whether the measured signal pattern itself has changed across interface conditions.

## Future Directions: Toward an Interface-Centered Evaluation Framework

The deformable and use-dependent nature of textile bioelectronic interfaces motivates a shift toward an interface-centered evaluation paradigm, in which representation stability is treated as a complementary evaluation criterion (Fig. 1). In this perspective, representation stability refers to the extent to which the structured pattern contained in, or derived from, a measured physiological signal remains comparable as the physical sensing interface changes. For sEMG, this structured pattern is termed a myoelectric representation. Myoelectric representation stability is therefore the extent to which comparable repetitions of the same motor task produce comparable myoelectric representations across changes in interface condition. This does not assume identical neuromuscular activation across repetitions. This concept draws on machine-learning ideas of robustness to distribution shift [9] and representation similarity [10–13]. The relevant lesson for textile bioelectronics is to evaluate the representation itself, not only the final output. This means testing how changes in the body–textile–electrode interface shape the myoelectric representation, making the interface itself a testable object.

**Fig. 1.**
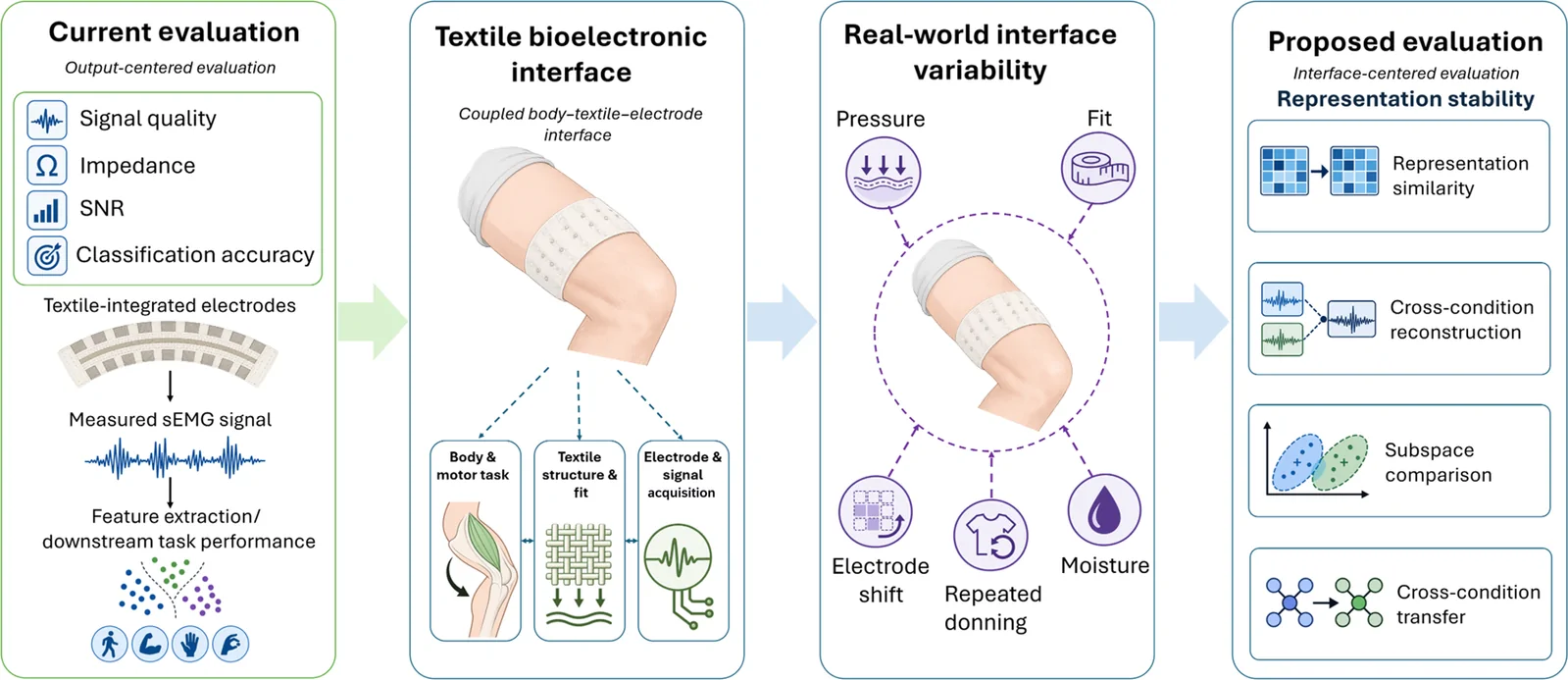
This perspective extends evaluation beyond signal quality and downstream performance by introducing representation stability as a complementary criterion for textile bioelectronic interfaces. From left to right, current evaluation is largely output centered, focusing on measured signal quality and downstream task performance. An interface-centered view treats textile bioelectronic systems as coupled body–textile–electrode interfaces, in which real-use variability in contact pressure, fit, moisture, electrode displacement, and donning configuration may alter the measured representation. Representation stability asks whether measured signal patterns remain comparable as the interface changes during real use.

On this basis, this perspective proposes an interface-centered framework for evaluating textile bioelectronic interfaces. Selected interface conditions such as fit, moisture, donning configuration, and electrode placement should be deliberately varied rather than treated only as unwanted variability. The resulting interface state should be measured, and representations should be compared both within and across interface conditions.

In practice, evaluating representation stability in sEMG first requires repetitions of the same motor task to be sufficiently similar, based on relevant kinematic or mechanical variables. Representation repeatability should then be characterized under a fixed interface condition to establish the normal level of within-condition variability. Once this reference has been established, representations obtained under deliberately altered interface conditions can be compared using an appropriate comparison measure and judged against a pre-specified, scientifically meaningful criterion for acceptable change rather than a universal cut-off [14,15]. Possible comparison measures include, for example, cosine similarity, centered kernel alignment, Procrustes-based comparisons, or subspace comparisons [10–14]. Beyond representation stability itself, it is also important to consider the downstream consequences of representation change. Cross-condition transfer provides one way to examine these consequences. An important question here is whether a signal basis or model learned under one interface condition transfers to another. Does a dominant signal basis learned under one pressure condition reconstruct data acquired under another? Does a classifier maintain performance because the representation is stable, or because it has learned to accommodate condition-specific changes? If 2 pressure conditions produce similar signal quality but different representations, a classifier trained under one condition may rely on condition-specific rather than task-related features. Conversely, if the representation remains stable across perturbations, the interface may better support cross-condition generalization and long-term use.

## Summary

Representation stability evaluation links engineering design with biomedical interpretation. In applications such as rehabilitation and home health monitoring, users will not reproduce laboratory interface conditions exactly from day to day. Understanding how those changes affect the representation could support more reliable interpretation outside controlled settings. For smart textile manufacturing, systematically characterizing the relationship between textile structure, body coupling, and signal representation could provide a basis for more systematic design rules, moving development from empirical iteration toward a more predictive design paradigm.

Understanding how interface variability affects representation stability is relevant to current textile bioelectronic sensing systems and may become increasingly important as the field expands [16,17]. The next step for textile bioelectronics is therefore not only to make better electrodes or more accurate classifiers. “Can we record and classify?” was the right question for feasibility. For real-world wearable bioelectronics, the sharper question is now: does the representation remain stable when the interface changes during real use?
